# Getting value out of working memory through strategic prioritisation: Implications for storage and control

**DOI:** 10.1177/17470218241258102

**Published:** 2024-07-31

**Authors:** Richard J Allen, Amy L Atkinson, Graham J Hitch

**Affiliations:** 1 School of Psychology, University of Leeds, Leeds, UK; 2 Department of Psychology, Lancaster University, Lancaster, UK; 3 Department of Psychology, University of York, York, UK

**Keywords:** Working memory, attention, prioritisation, value, reward

## Abstract

Working memory is an active system responsible for the temporary maintenance and processing of information in the support of cognition and action. In keeping with this, a growing body of research has explored the close links between working memory and attention, and how these might be harnessed to impact performance and possibly improve working memory efficiency. This is theoretically and practically important, given that working memory is a central hub in complex cognition yet is extremely capacity- and resource-limited. We review work carried out over the last 10 years or so looking at how high “value” items in working memory can be strategically prioritised through selective attention, drawing principally from visual working memory paradigms with young adult participants, while also discussing how the core effects extend to different task domains and populations. A consistent set of core findings emerges, with improved memory for items that are allocated higher value but no change in overall task performance, and a recency advantage regardless of point allocation when items are encountered sequentially. Value-directed prioritisation is effortful, under top-down strategic control, and appears to vary with perceptual distraction and executive load. It is driven by processes operating during encoding, maintenance, and retrieval, though the extent to which these are influenced by different features of the task context remains to be mapped out. We discuss implications for working memory, attention, and strategic control, and note some possible future directions of travel for this promising line of research.

Working memory is a critical point of convergence between perception, long-term memory (LTM), and action, and is closely linked to attention (Baddeley, 2012; Baddeley et al., 2021). The multi-component model of working memory introduced by Baddeley and Hitch (1974) and developed in subsequent iterations (Baddeley, 1986, 2000; Baddeley et al., 2021) describes a limited-capacity system that plays a central role in complex cognition. As with other broad theoretical frameworks of working memory (e.g., Barrouillet & Camos, 2021; Cowan et al., 2021; Mashburn et al., 2021), attentional control is integral. This is in keeping with the suggested position of working memory as an interface between what Chun et al. (2011) label as external and internal attention. Optimal task performance depends on the ability to apply attentional control in a way that effectively holds in mind task-relevant information in an appropriate and accessible form and suppresses unwanted information.

There are various ways in which attention can be directed within working memory. One prevalent method (discussed later in this review) has been to indicate via a visual or other perceptual cue which item in a memorised set is most likely to be tested. This method of directing attention can be implemented before, during, or after target encoding, and results in performance enhancements relative to uncued items or neutral conditions where no item is cued (Griffin & Nobre, 2003; Souza & Oberauer, 2016). An alternative method that has emerged relatively recently is to encourage strategic prioritisation of certain items through allocation of differential rewards for correct responses (e.g., point values; see next section for details). In this approach, all items are tested equally often. This value-guided method of directing attention has started to yield novel insights regarding the relationship between working memory and attention, interactions between perceptually driven and internally controlled attentional selection, and the importance of considering strategic approaches when exploring working memory from both theoretical and applied perspectives. The present review offers an overview of research on this method of examining strategic prioritisation over the last 10 years, placing it in context, evaluating some of the insights we might derive concerning working memory, selective attention, and strategic control, and signposting where the area might go next. We begin with an overview of the core findings associated with value-driven prioritisation that has emerged across different studies, before considering possible interpretations and insights that can be drawn from this and ancillary observations.

## Value-directed prioritisation of items in working memory

**Figure 1.**
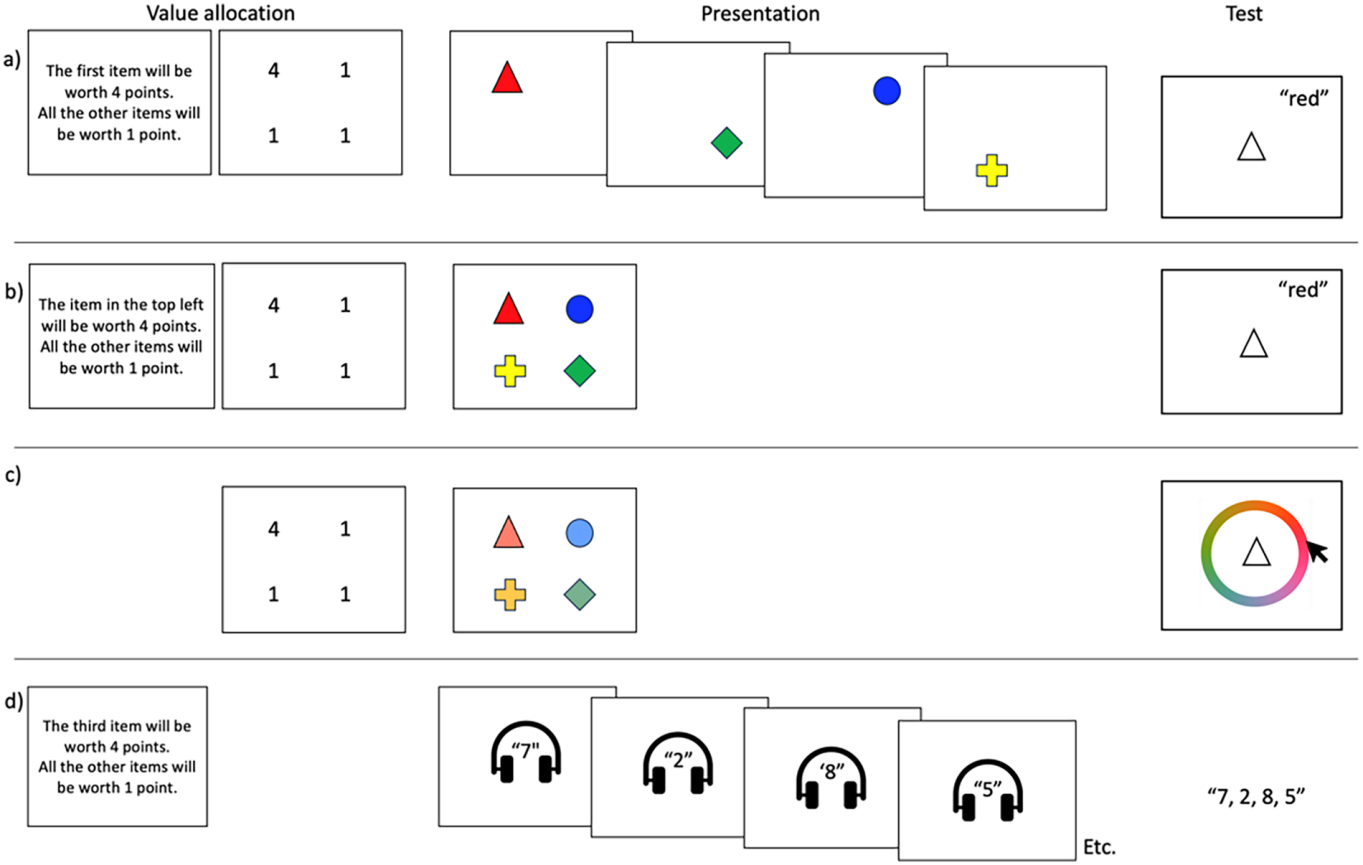
Schematic illustration of selected point allocation methodologies implemented across different paradigms to date, showing (a) a sequential visual task and cued recall, (b) a simultaneous visual task and cued recall, (c) a simultaneous visual task and continuous response, and (d) a sequential auditory-verbal recall task for digit sequences with serial recall. Points can be allocated via instruction provided at the start of a test block, or (for visual paradigms) via values presented in locations on the screen (using shifting distributions between trials). See Table 1 for more details and example studies.

This approach was first adopted in a series of experiments reported by Hu et al. (2014) that built on earlier studies exploring memory for visual feature bindings (Allen et al., 2006, 2012). Coloured shapes were briefly presented in a sequence (see Figure 1a), followed by a verbal cued recall test for one of the sequence items. Point values were assigned to each item, and participants were told they would earn if they were tested on that item and they responded correctly. In different blocks of trials, more points were offered for either early or late items in the sequence. For example (Hu et al., 2014, Experiment 4), in one set of trials the first item presented was worth four points, while other items were each worth one point. In another set of trials, the final item was worth four points and the rest were worth one point. Importantly, and unlike visual cueing studies, these points were not predictive of test probe frequency, with every item equally likely to be tested. Point values had no prior association with any of the to-be-remembered stimuli and were also not associated with any tangible reward (monetary or otherwise).

Using this approach, performance profiles across the sequence were clearly affected by point distributions; a larger recency effect was apparent when later sequence items were more valuable, and a clear primacy effect emerged when higher value was allocated to early sequence items. Thus, participants were clearly able to respond to the differential distribution of value by strategically prioritising items that were in more “valuable” serial positions. This emerged in the context of primacy–recency asymmetry, with an uplift in accuracy for recent items even when a high value was allocated to the primacy portion of the sequence, reflecting an automatic component to this effect (Allen et al., 2014).

**Figure 2.**
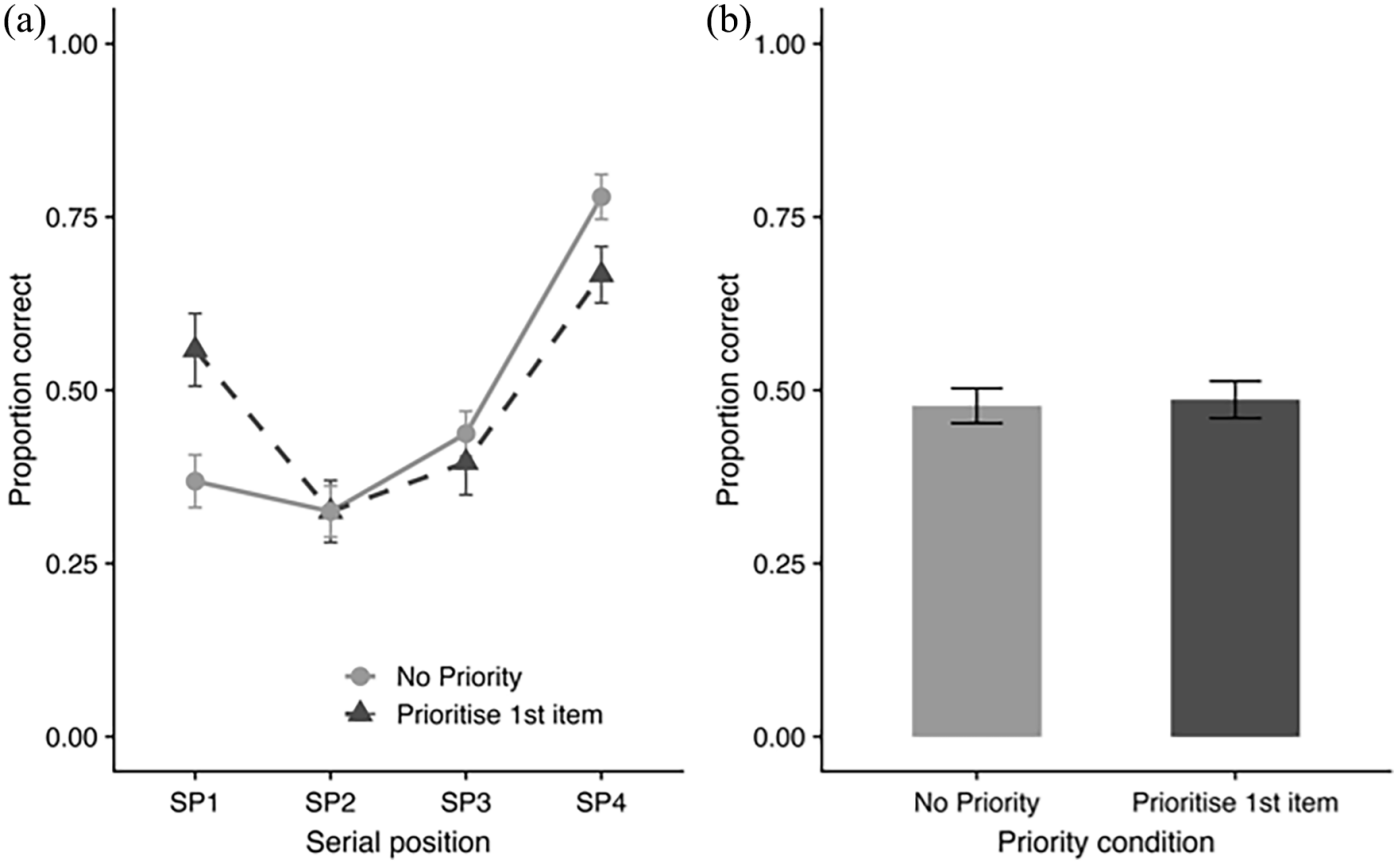
Illustration of recall accuracy in equal value and prioritisation conditions using sequential item presentation. (a) Performance as a function of serial position. (b) Overall accuracy in each condition. Data taken from Hitch et al. (2018, Experiment 2).

The observation of enhanced working memory accuracy for high-value items has since been replicated multiple times, across a range of task contexts. For example, the studies by Hitch et al. (2018) and Atkinson, Berry, et al. (2018) added an equal-value condition in which all items were allocated the same points, to distinguish between the possible gains of prioritisation (i.e., equal vs. high-value items) and the costs of deprioritisation (i.e., equal vs. low value). Relative to the equal value condition, prioritisation resulted in large gains for the high-value item and smaller costs for some of the low-value items (see Figure 2a). Hitch et al. (2018) also demonstrated that the value effect can generate performance improvements at any position in the sequence.

This trade-off between a performance enhancement for the high-value items and a performance decrement for low-value items highlights an important observation that we find across different tasks, namely that value/reward-based prioritisation effects are item-specific and do not manifest as overall changes in performance levels (see Figure 2b). Overall performance for trials where a higher value is allocated to a particular item typically does not differ from when all items are of equal value (e.g., Allen et al., 2021; Atkinson, Berry, et al., 2018; Atkinson et al., 2019; Hitch et al., 2018). Similarly, performance on trials where an earlier item in the sequence is more valuable does not differ from performance on trials where a later item in the sequence is more valuable (Hu et al., 2014, 2016). These patterns indicate that value does not serve to increase working memory capacity. Instead, limited-capacity storage and processing resources are flexibly shifted between items based on their allocated value.

### Extending value effects across different contexts

**Figure 3.**
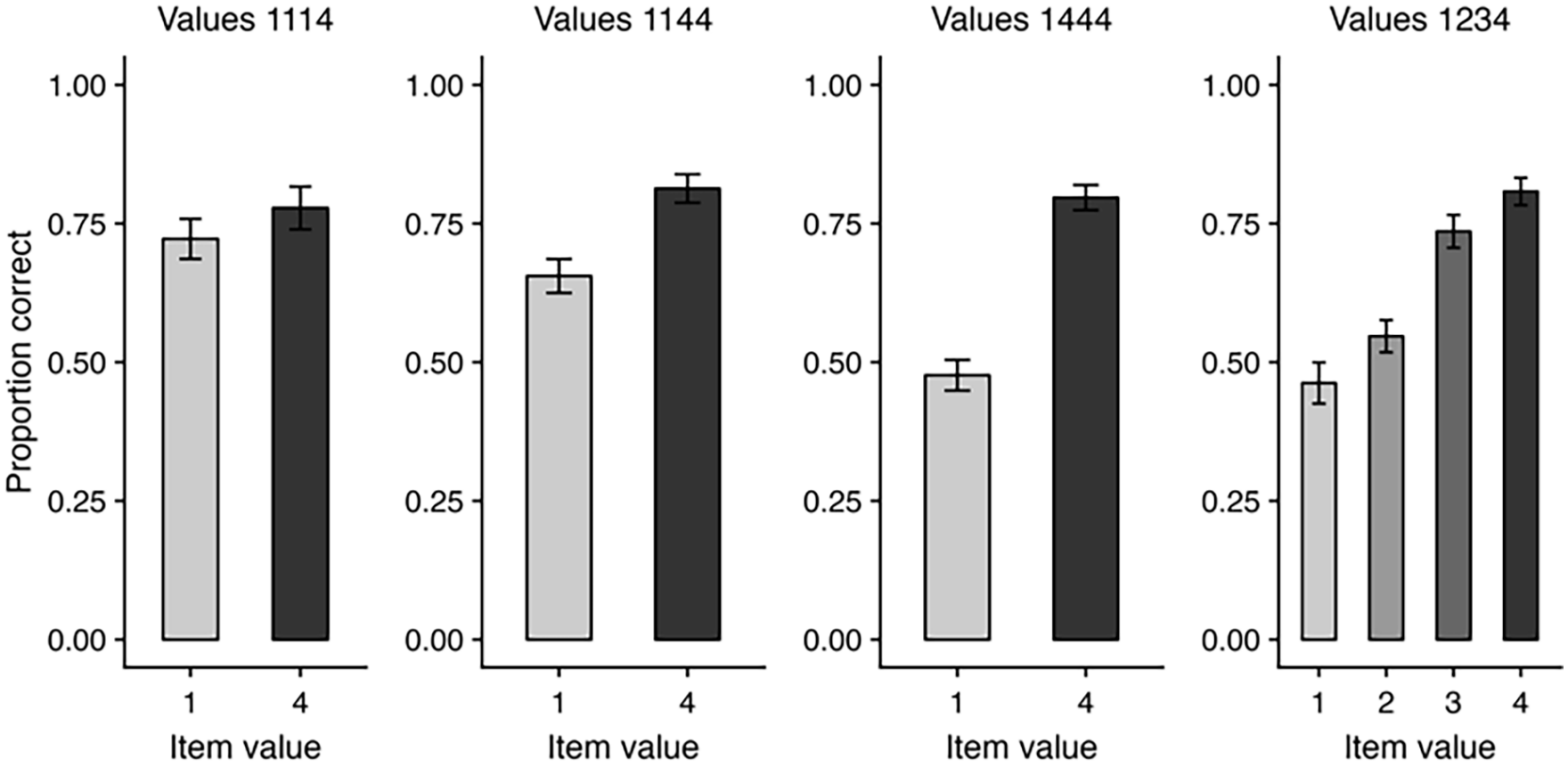
Recall accuracy in prioritisation conditions (either with 1–3 high-value items, or values ranging from 1 to 4 within each display) with simultaneous item presentation. Data taken from no-suffix trials in Allen and Ueno (2018, Experiments 1–4).

Research to date has often presented items sequentially and asked participants to prioritise items located at certain points in the sequence, enabling analysis of cued recall accuracy across different serial positions. It is also possible for value-driven prioritisation to be applied to items from within simultaneously presented multi-item arrays. Here, value is typically allocated to items on a spatial basis (see Figure 1b). Using this approach, Allen and Ueno (2018) observed greater recall accuracy for high-value than low-value items within simultaneously presented four-item displays, though effects were clearer when multiple items were allocated with higher value (e.g., three high-value items and one low-value item); much smaller value effects were observed with only one high-value item (and three low-value items) in the array (see Figure 3). It is likely that, as multiple stimuli can be prioritised when encountered simultaneously, the presence of only one high-value item increases the likelihood that participants spontaneously prioritise some of the low-value items too, thus washing out the value effect that can be observed. Allocating high value to multiple items in the array means that any low-value items are much less likely to be prioritised, although they are not completely neglected or discarded from working memory as performance is still well above chance. Note that overall accuracy levels were similar across these shifts in value ratios (for no-suffix data as in Figure 3, proportion correct was .74 for the 1114 experiments, .73 for the 1144 experiments, and .72 for the 1444 experiments), in keeping with constant overall capacity limits found with sequential presentation. Finally, a graded pattern of recall accuracy was found when different items were allocated values ranging from 1 to 4 points (Figure 3), indicating an impressive degree of control in how attention can be strategically allocated across items in the environment.

Atkinson et al. (2022) employed a similar approach of spatially allocated value across a simultaneously encountered four-item display, but rather than using a categorical verbal cued recall measure as employed by previous studies, this study instead adopted a continuous response task. The task still required memory for shape–colour conjunctions, but participants had to precisely reproduce the associated colour on a colour wheel in response to a shape cue. Convergent value effects were observed in this task, with lower recall error for high-value items. Mixture modelling can be applied to such data, which yields the probability of recalling the target item (e.g., the tested item), the probability of recalling a non-target item (i.e., a non-tested item), and precision (the fidelity of the representation; Bays et al., 2009; Oberauer et al., 2017). Mixture modelling conducted on this data revealed that the probability of recalling the target item was higher for high-value items relative to equal and low-value items, while the probability of recalling a non-target item was lower. Precision was also higher for high-value items relative to low-value items. Value effects have also been found on a continuous response measure of colour-orientation binding within a sequential working memory task (Hu et al., 2023). Finally, several studies have implemented recognition at the test phase, again demonstrating clear value effects on working memory performance (e.g., Atkinson et al., 2024; Sandry & Ricker, 2020). Thus, using notional value as a tool to encourage strategic prioritisation of certain items yields observable impacts across a range of response methods typically used in working memory tasks. In each case, we see enhanced memory for high-value items alongside some reduction in low-value items and no overall difference in performance, along with recency effects when items are encountered sequentially.

As with the broad literature using attentional cueing, research on value-directed prioritisation in working memory has tended to focus on visual memory and in doing so attempts to control and minimise contributions from other domains and modalities (e.g., verbal processing and storage). However, it is of theoretical and practical importance to explore how such effects might generalise beyond visual working memory. Finding evidence for generality would illustrate that this approach taps into processes of attentional allocation that are broadly applicable across working memory and not limited to specific domains or experimental paradigms. First, there is evidence that visually presented verbal material can be strategically prioritised (Sandry et al., 2014, 2020). In this paradigm, sequences of three lower-case letters or words were presented on screen, with the high-value target denoted by presentation in a different font colour. Subsequent recognition of a target versus a foil (using upper case presentation) was superior for higher value words compared with low value or an equal value condition, a pattern that emerged regardless of the serial position of the higher value item. Similarly, Labaronne et al. (2024) found that visually presented words (encountered within a cognitive load paradigm alongside a number parity task) were better recalled when associated with a higher (monetary) versus a lower value. However, although verbal in nature, these studies presented material within the visual modality. In addition, the association of prioritisation with visual appearance (Sandry et al., 2014, 2020) or monetary reward (Labaronne et al., 2024) means that other factors may be at work in the effects observed.

As a stronger test of whether value effects extend to other modalities, we implemented a direct test of auditory–verbal prioritisation using a verbal serial recall task (Atkinson, Allen, et al., 2021). Digit sequences were presented in spoken form, with values either equal across all items or higher at a certain serial position (e.g., at the third, fifth, or seventh position in a nine-digit sequence). In contrast to work in the visual domain, participants were asked to recall the entire sequence in order. Alongside standard primacy and recency effects, recall was significantly enhanced for the items that were of higher value, with some reduction in accuracy observable for other items in the sequence. Thus, strategic prioritisation is indeed possible in auditory–verbal working memory. Similarly, recent work suggests that value-directed prioritisation can also be applied to cross-modal bindings of visual and auditory features (Cinar et al., in preparation). Here, participants were able to show enhanced recall for the first visual–auditory pairing in a sequence when it was allocated a higher value, alongside no overall change in performance compared with an equal-value condition.

Further generalising to another modality, Roe et al. (2024) adapted the methodology of Atkinson, Allen, et al. (2021) and applied it to a tactile memory paradigm. Touch was applied to different digits on the participant’s hand (with visual input removed), followed by the requirement to reproduce the sequence using finger movements (Johnson et al., 2016; Roe et al., 2017). The core findings from other modalities were replicated within this context, with improved accuracy for high-value items and costs to low-value items from the same sequence relative to an equal-value condition, a trade-off that resulted in no overall main effect of the value condition. A second experiment ruled out an explanation that solely attributed such effects to verbalisation, with the patterns surviving under concurrent articulatory suppression (AS). Thus, prioritisation appears to operate in a functionally analogous way across visual, verbal, and tactile modalities, with performance enhancements to high-value items, some decrement to low-value items, and no overall change in capacity.

Finally, a study by Johnson and Allen (2023) applied the standard cued recall methodology used in studies of visual (shape–colour) prioritisation (e.g., Hu et al., 2014) to a novel task assessing binding between colour and odour. A series of odours contained within different coloured containers were presented, followed by a probe odour within a neutral-coloured container at the test, with participants required to verbally recall the associated colour. A recency effect was found, along with a significant shift towards primacy when the first item was allocated with a higher value. The numerical advantage for the high-value item over the same serial position in the equal-value condition was not statistically significant, suggesting that strategic prioritisation might be less effective in this context, though it remains to be seen whether this reflects difficulty with olfactory processing specifically, or with any form of non-unitised binding in which features are encountered in disparate forms.

## Interaction with executive and perceptual interference

So far, we have seen a value-driven prioritisation effect that consistently emerges across different task contexts. In trying to understand the core effect, it is useful to identify possible strategic and non-strategic boundary conditions for reliable observation of this effect. One category of condition might be the availability of general attentional control resources, connected to the concept of central executive control in working memory (Baddeley, 1986, 1998). In the first study to address this question, Hu et al. (2016) examined sequential colour-shape binding and single-probe recall, applying a dual-task manipulation in which the participant concurrently performed a verbal task that was simple (repetition of numbers) or somewhat more attention-demanding (backward counting). The size of the value effect at either the first or last sequence position was reduced or abolished when performing the more demanding task. At the same time, a strong recency effect was still observed in all conditions, illustrating an automatic aspect of this latter component. The same question was tackled in a different way by Atkinson, Allen, et al. (2021) using verbal serial recall of digit sequences, with simple or more attention-demanding concurrent tasks applied to value-driven prioritisation and equal-value control conditions. In this case, the high-value advantage was not diminished under increased executive load. Instead, participants were only able to recall (above levels expected by chance) the high value and the last item in the sequence under these conditions.

A combination of factors may be at work here. First, general attentional resources may be more critical for prioritisation in visuospatial (i.e., Hu et al., 2016) relative to auditory–verbal (Atkinson, Allen, et al., 2021) tasks. Second, test method and probability may also be an important dimension to consider (see Table 1), in that the probability of a high-value item being required at the test is lower for single-probe cued recall (Hu et al., 2016) than for serial recall of all items (Atkinson, Allen, et al., 2021). This may impact how participants choose to strategically allocate their limited resources around the task space, particularly when these resources are further constrained by a demanding concurrent task. In either case, we assume that effective prioritisation draws on attentional control resources, but its implementation reflects endogenous strategic control and the motivational influence of task context. This shifting prioritisation effect stands in contrast to the continuing presence of a substantial recency effect for late-sequence items regardless of executive load.

**Table 1.** Summary of methodological details in value-directed prioritisation paradigms and example studies.

Dimension	Details	Example study
***Prioritisation*** High value items Value comparisons Value allocation Allocation method Test probability Value ‘reward’	1 per trial More than 1 per trial High vs. low High/low vs. equal Graded value Blocked and consistent Varying, pre-encoding Varying, post-encoding Verbal instruction Spatial configuration Item appearance Equal probability Increased for high value All items tested Notional Gamified context Monetary reward	Most studies (e.g. Hu et al., 2023) Allen & Ueno (2018); Hitch et al. (2018) Hu et al., (2014), Allen & Ueno (2018) Atkinson, Berry, et al., (2018), Allen et al. (2021) Allen & Ueno (2018), Hu et al. (2014) Hu et al. (2014), Hitch et al. (2018) Allen & Ueno (2018), Atkinson et al. (2022) Allen & Atkinson (2021), Jeanneret et al. (2023) Hu et al. (2014), Atkinson et al. (2018) Allen & Ueno (2018); Atkinson et al. (2022) Sandry et al. (2014, 2020) Most studies (e.g. Hu et al., 2014) Atkinson, Berry, et al. (2018) Atkinson et al. (2021); Roe et al. (2024) Most studies (e.g. Hu et al., 2014) Atkinson et al. (2019) Brissenden et al (2023); Zheng et al. (2022)
***Task context*** Item presentation Test method Modality	Sequential Simultaneous Single recall Single recognition Single continuous Serial recall of all items Visual Visual-verbal Auditory-verbal Visual-auditory Visual-olfactory Tactile	Hu et al. (2014); Atkinson, Berry, et al. (2018) Allen & Ueno (2018); Atkinson et al. (2022) Allen & Ueno (2018); Hu et al. (2016) Atkinson et al. (2024); Sandry & Ricker (2020) Atkinson et al. (2022); Hu et al. (2023) Atkinson et al. (2021); Roe et al. (2024) Most studies (e.g. Hu et al., 2014) Sandry et al. (2014, 2020) Atkinson et al. (2021) Cinar et al. (in preparation) Johnson & Allen (2023) Roe et al. (2024)

A second possible limiting factor is the degree of stimulus-driven, exogenous perceptual interference that is present in the environment. One way of examining this is through a presentation of a to-be-ignored suffix stimulus shortly after target offset, which draws features (colour and shape) from the experimental stimulus pool that are not being used on that specific trial (Ueno, Allen, et al., 2011; Ueno, Mate, et al., 2011). Several studies have shown that a suffix serves to reduce or abolish the recency advantage for late-sequence feature combinations (Hu et al., 2014, 2016, 2023; Hitch et al., 2018). Furthermore, these studies also found that recall for items assigned with higher value declined with a post-presentation suffix, while low-value items were less affected (Hitch et al., 2018; Hu et al., 2014, 2016), suggesting the vulnerability of prioritised information in this context. Allen and Ueno (2018) also observed increased suffix interference for high (compared with low) value items using the simultaneous presentation of the target array, though this was only apparent when multiple items were allocated with higher values. However, at this point we would note evidence that reward does not always reliably induce vulnerability (e.g., Hu et al., 2023; Vergauwe et al., 2023; Zhang & Lewis-Peacock, 2023), suggesting an interaction that may be limited in its generalisability, though there is no evidence that higher value information is *protected* from interference.

Thus, recency and value-based prioritisation provide benefits for item accessibility, but this does not protect against subsequent perceptual interference and might even come with a cost in terms of vulnerability at least in certain contexts. This is analogous to recent suggestions that items in an active state in visual working memory are more vulnerable to interference (Lout et al., 2023). These findings also show how strategic and stimulus-driven attention might interact when they point in different directions. The suffix has visual features that match the top-down instruction *remember* and a temporal feature that matches the top-down instruction *do not remember*. This conflict is reflected in the tendency for the suffix to be reported as an intrusion error in recall. Indeed, it may be useful to consider the degree and type of conflict that an interfering stimulus introduces into the task set. Increased perceptual interference for more recent and higher value items has been observed using a suffix that participants are instructed to ignore (Allen & Ueno, 2018; Hitch et al., 2018; Hu et al., 2014, 2016) In line with this, Zhang and Lewis-Peacock (2023) found that prioritisation (elicited through predictive retro-cueing) increased vulnerability to subtle distractor-oriented distortion when the task context required minimal engagement with the distracting stimulus. Prioritisation protected against full displacement of the memory representation when the task required more engagement with the distractor. Thus, the extent and type of processing applied to perceptual input can determine the form of interference that then arises.

To sum up these findings then, recency effects using sequential presentation remain under executive attentional load but appear to be reduced by retroactive perceptual interference. In contrast, there is some evidence that prioritisation effects can shift with attentional load and perceptual interference, reflecting the complex interplay between strategic direction and different forms of attentional control.

### Prioritisation across the lifespan

Most research on this topic has been conducted with typical young adult participants, with the aim of establishing the profile of value-based prioritisation effects and how these interact with a range of experimental factors. It is important both from theoretical and applied perspectives to explore to what extent the core findings generalise to different developmental populations. This would be in keeping with how our understanding of working memory has benefitted by drawing on convergent evidence from multiple populations. For example, Hitch and Halliday (1983; see also Hitch, 2002) discussed the value of developmental evidence for informing working memory (see also Cowan, 2023), while the multi-component working memory model has often been linked to evidence from healthy ageing and neuropsychological populations (e.g., Baddeley et al., 2010, 1991). Effortful and conscious attentional control may progressively develop through childhood and later decline with healthy ageing, in contrast with more automatic processes (e.g., Zelazo et al., 2004). Relatedly, proactive control and metacognition improve from childhood to adulthood (Chevalier et al., 2014; Forsberg et al., 2021), and may decline with age (Ball et al., 2023; Palmer et al., 2014). These are each likely to be important factors in determining whether an individual can successfully implement strategic control to enhance task performance. Evidence that prioritisation effects may or may not vary with broader changes in lifespan cognition, therefore, has implications for selective attention and working memory, and for developmental cognition, and can cast light on whether instructed prioritisation might offer viable future routes to optimise working memory in a practical sense.

Our first foray into this question with a developmental population (Berry et al., 2018) applied a version of the task and set-up that was closely based on the paradigm commonly implemented with young adults, albeit reducing sequence length to three items to make the task more suitable for children. Across three experiments, children aged 7 to 10 years reliably produced a last item recency effect but showed no sign of prioritising an item of higher value in the sequence (either to the first or final sequence position). Thus, while the children were able to produce an automatically derived (e.g., Allen et al., 2014) recency advantage, the null effect of item value at first glance seemed to suggest that they typically lacked the cognitive control to support strategic prioritisation.

**Figure 4.**
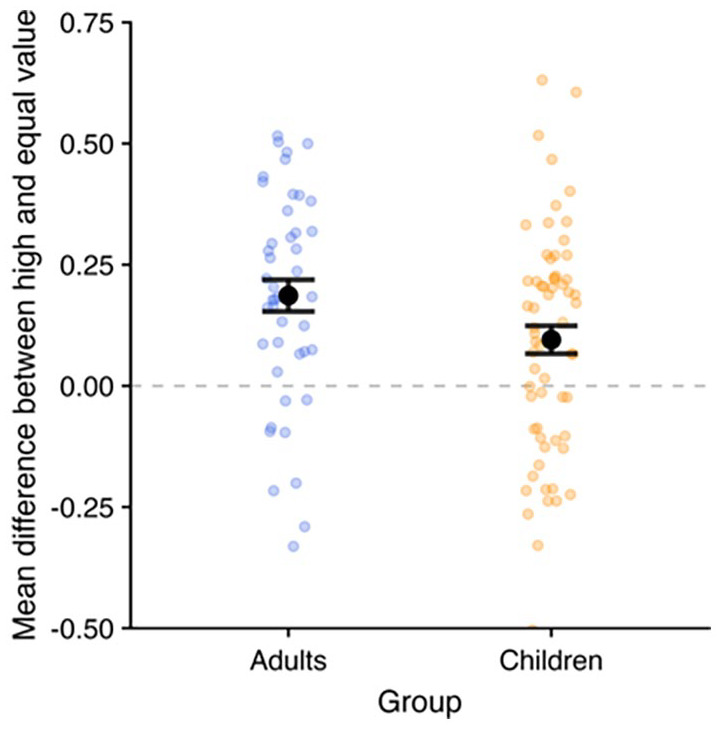
Data from adults (Atkinson et al., 2018, Experiment 1) and children (Atkinson et al., 2019, Experiment 1), showing the mean difference between high-value and equal-value trials at the prioritised sequence position for individual participants as well as the group mean (and *SE*). Data are drawn from comparable conditions (using the same set sizes and timings), and the dotted line indicates no priority effect.

This possibility was further explored by Atkinson et al. (2019) in a study that embedded the same basic paradigm into a gamified task context designed to be more child-friendly and enhance the meaning and accessibility of the critical point value manipulation. Children aged 7 to 10 years were introduced to a friendly alien named “Zorg” and told that they must help him collect “energy points” that would determine how long they would have to play a short post-task alien-themed game. A progress bar showing their apparent cumulative points score was interspersed between trials, though this was purely for motivational purposes and did not indicate genuine performance. In the first experiment using three and four-item sequences, children showed the recency advantage as seen in Berry et al. (2018), but now they also produced an advantage for high-value items at the first serial position, relative to an equal value condition. The effect did not differ across 7 to 8-year-old and 9 to 10-year-old children, although it was somewhat smaller at a group level than that typically seen in young adults (e.g., Cohen’s *d* = .42 in Experiment 1 vs. *d* = .86 in Atkinson et al., 2018, using comparable conditions), with more children failing to show the benefit (see Figure 4).

A second experiment shifted from sequential to simultaneous presentation, and again found that children produced a recall advantage for high-value items, though this was conditional on memory load, in that children only demonstrated evidence of prioritisation for four-item displays (*d* = .65), and not for three items (*d* = .19). There was no overall effect of value in either experiment, providing evidence of a resource trade-off as observed in adults (e.g., Atkinson et al., 2018), though costs to low-value items (compared with equal value trials) were not always significant. Thus, children can direct attention based on reward in working memory when they are particularly motivated to do so. However, in line with evidence from visual cueing (e.g., Shimi et al., 2014; Shimi & Scerif, 2015, 2022), effects at the group level appear to be somewhat smaller than those typically observed in adults, possibly reflecting developmental improvements in executive function (Diamond, 2013), selective attention (Astle et al., 2012), and proactive control (Chevalier et al., 2014).

It is also important to examine developmental changes in later life, given that healthy ageing is typically associated with a decline in executive functioning, working memory (Brockmole & Logie, 2013; Hedden & Gabrieli, 2004; Johnson et al., 2010; Park et al., 2002; Swanson, 2017), and possibly proactive control (Ball et al., 2023). Ageing may impact both the formation and the subsequent active maintenance of working memory representations (Slana Ozimič & Repovš, 2020). Within the visual cueing literature, there is some evidence that older adults can experience similar-sized cueing effects to younger adults (Gilchrist et al., 2016; Loaiza & Souza, 2018; Mok et al., 2016; but see Duarte et al., 2013; Newsome et al., 2015), though may struggle to preserve these benefits against distraction (Loaiza & Souza, 2019). We administered a visual working memory task involving sequences of three coloured shapes to young and older adults, applying higher value to each of the serial positions (Experiment 1) or specifically to the mid-sequence position (Experiment 2), in each case comparing performance against an equal-value condition (Allen et al., 2021). Although older adults were somewhat less accurate at the task overall, they produced a recall advantage for high-value items that were at least as large as that seen in their younger counterparts. As repeatedly demonstrated with young adults across different studies, the older group also exhibited some costs to low-value items and showed no overall change in performance. At least based on this initial evidence then, strategic attentional direction seems to be intact in the broader context of age-related decline in visual working memory and executive control. This fits with a finding reported by Atkinson, Baddeley, et al. (2018) that older adults showed the same performance benefit as younger participants when encouraged to focus on an unspecified subset of items rather than the whole array. Value-directed prioritisation may provide a useful practical way forward in helping older adults marshal their available cognitive resources to optimise working memory task efficiency.

## Implications for working memory and attention

A body of evidence now shows that items allocated with a higher value can be prioritised with beneficial effects on working memory for these items, alongside costs to other less valuable items and no overall change in performance. Value-directed prioritisation appears to provide not only a novel way of exploring the relationship between storage and attention but also a way of pulling apart their contributions to working memory performance. Attentional control can shift focus between items, but total storage capacity remains fixed. We might think of these results as representing two forms of capacity, one for storage and one for attention, each limited but in different ways and with different implications for performance. Limits on storage capacity would constrain how many items can be effectively held overall, and limits on attentional control constrain what can be effectively prioritised at any time. At this point, we would note the ongoing debate, principally in the visual domain, regarding whether working memory capacity is limited by the number of slots and or the resource pool available for processing (Bays et al., 2009; Zhang & Luck, 2008), although our approach is ultimately agnostic regarding this debate and does not critically hinge on either position. Regardless, the functional outcome is that given WM performance is typically highly constrained, attentional control is important in optimising performance under these constraints, and the selected items are enhanced through prioritisation, but overall capacity generally is unaffected.

We have broadly interpreted the basic observation of enhanced memory for high-value items as reflecting storage in a highly accessible state within working memory. High-value information is more likely to be in this privileged state, relative to information of lower value. This represents active and consciously controlled operations at a modality-general level, given that effects have been found within tasks that target different domains and modalities. We have mapped this state of heightened availability within conscious awareness onto the episodic buffer component within the multi-component working memory framework (Baddeley et al., 2021; Hitch et al., 2020). The episodic buffer is described as a modality-general storage and processing capacity, providing a consciously accessible point of convergence between different forms of modality-specific input, LTM, and action. It was introduced into the multi-component model by Baddeley (2000) as a way of broadly capturing how these important cognitive dimensions might interface within working memory. This offered potentially greater explanatory power, given that the original tripartite model of Baddeley and Hitch (1974; Baddeley, 1986) did not explicitly address such questions, though with a trade-off against parsimony (Andrade, 2002). It also offered more common ground between the multi-component model and Cowan’s embedded processes approach (Cowan, 1999; Cowan et al., 2021), as there are clear similarities between the episodic buffer and the concept of a focus of attention (FoA) within working memory as described in the embedded processes framework (see, e.g., Baddeley, 2012; Gray et al., 2017; Hitch et al., 2020). The FoA is a useful concept in the context of the present work, and so we use it when referring to the storage of one or more items in a state of enhanced accessibility and awareness, though explicitly mapping it onto the episodic buffer within the multi-component model.

What enters and remains in the FoA within working memory reflects both stimulus-driven, externally motivated, bottom-up influences, and internally motivated top-down control. Such a distinction has a long history in the context of selective attention during perception (Broadbent, 1958; Kahneman et al., 1992; Lachter et al., 2004; Treisman, 1960, 1986; Treisman & Gelade, 1980; Yantis, 2000). Automated processing and top-down control are also key features of the attentional framework described by Norman and Shallice (1986) that incorporate the supervisory attentional system. This approach heavily informed Baddeley’s (1986) description of the central executive, a set of control resources incorporated into the multi-component framework (Baddeley, 2012, 1986; Baddeley & Hitch, 1974; Baddeley et al., 2021; Hitch et al., in press) and other accounts of working memory (Barrouillet & Camos, 2021; Cowan et al., 2021). Top-down executive control of attention may be central to the predictive power of working memory for fluid intelligence and a host of other real-world abilities and attainments (Draheim et al., 2022; Shipstead et al., 2016). The critical role of the central executive in supporting task performance highlights the importance of *working* memory (rather than passive short-term memory [STM]) in complex cognition, underlining one of the original principles of the multi-component approach (Baddeley & Hitch, 1974).

The “central executive,” being a collective term for what is likely a range of executive functions, has been acknowledged as a “conceptual ragbag” and a homunculus (Baddeley, 1986, 2012) and even earmarked for retirement (Logie, 2016). We would argue that it still serves a useful purpose as an umbrella term for a set of resources, and evidence indicates some functional unity as well as diversity (Duncan et al., 1997; Miyake et al., 2000). Fractionation has been suggested along several dimensions. Along with functions such as task switching and retrieval from LTM, Baddeley (1986, 1996) suggested the ability to focus and divide attention as being core aspects of central executive control. Guiding attention to goal-relevant information during encoding, consolidation, maintenance, and retrieval, and identifying and implementing strategies to optimise task performance, may also be important (Baddeley et al., 2021) and are likely critical in enabling stimulus prioritisation.

**Figure 5.**
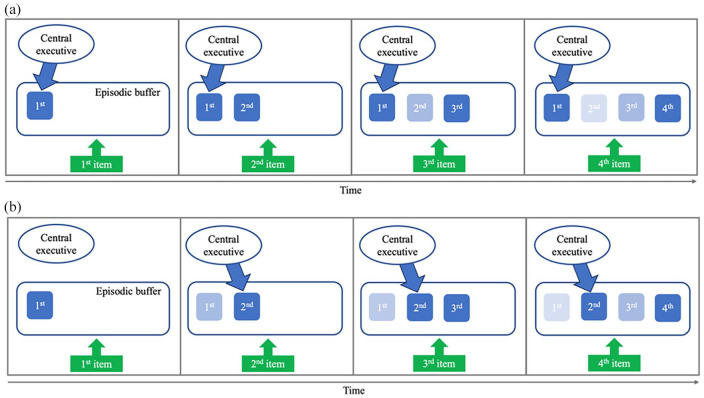
Illustration of encoding and maintenance of a four-item sequence in working memory, when either (a) the first item or (b) the second item is allocated with a higher value. Each presented item automatically enters the episodic buffer/focus of attention. It is then likely to be displaced by subsequent items. An item of greater priority can be strategically and actively maintained via executive control. Shading represents the probability of an item being available and accessible in the episodic buffer/focus of attention (with darker items more likely to be in this state).

We therefore assume that bottom-up, perceptually driven environmental input interacts with goal-directed, internally motivated, top-down attentional control that underlies value-driven strategic prioritisation. To achieve an advantage for a high-value item, the individual must consciously and strategically direct their attention towards this information during encoding, maintenance, and/or retrieval, normally at the expense of other stimuli that form part of the same task set. When a salient display of multiple items is encountered in the environment, at least some of this perceptual input will automatically capture attention and be encoded into working memory. Similarly, as a sequence of task-relevant stimuli is encountered, each perceptual input is likely to capture attention and lead to at least temporary registration in working memory (Maxcey-Richard & Hollingworth, 2013), provided consolidation is not disrupted. Each new input will then be briefly accessible within the episodic buffer/FoA, and this happens relatively automatically (see Figure 5). This process contributes to the recency effect in single-probe working memory tasks, a relative advantage that remains under increased executive load (Allen et al., 2014) even when later sequence items have low value (e.g., Atkinson, Allen, et al., 2021; Hu et al., 2016). Within this context, although salient exogenous information will be likely to capture attention and result in working memory encoding, stimuli can be strategically prioritised for encoding through direction of selective spatial attention towards those stimuli.

Thus, when value is allocated in time for encoding, the individual can choose to fixate on high-value stimuli, more so than information that is of lower value. Within the visual attention literature, the Guided Search model (Wolfe, 2021) identifies value as a form of prioritisation that can guide attentional allocation, and we assume similar principles would apply here. Indeed, analogous effects can be seen in visual attention tasks, where top-down prioritisation can improve the tracking of high-priority items (Crowe et al., 2019; Hadjipanayi et al., 2022) while not enhancing overall performance. This reflects how the strategic direction of externally oriented attention can then influence working memory. There is more to working memory encoding than simply attending to an item, however, with necessary perceptual attention but perhaps not always sufficient for working memory encoding (Oberauer, 2019). Intention to encode may be important, and encoding strategies that favour retention of high-value items are likely to be adopted, depending on the individual and what the task context allows. Focusing attention on an item during encoding then may serve to optimise effective consolidation into working memory (Ricker et al., 2018).

Following encoding, strategic maintenance within the FoA may be achieved in part through attentional refreshing (Figure 5). This has been described as a domain-general process of keeping mental representations in an active and accessible state through the application of attention (Barrouillet et al., 2004; Camos et al., 2018; Johnson, 1992; Raye et al., 2002), and has been connected to value-directed prioritisation effects (Atkinson et al., 2022; Sandry et al., 2020). Refreshing prioritised items may help to stabilise the memory and ensure it remains active and accessible. Atkinson et al. (2022) combined the value manipulation with a directed refreshing methodology (Souza et al., 2015) in which a cue is presented post-encoding, asking participants to think of (i.e., refresh) one of the previously presented stimuli. Value and cueing effects interacted; cueing participants to refresh an equal or low-value item enhanced performance, whereas cueing participants to refresh a high-value item had no effect. This indicated that the high-value item was already being refreshed, meaning that cueing it for directed refreshing became redundant.

Attentional refreshing could involve a cycling of covert retrieval (Camos et al., 2018) that is biased in duration and/or frequency towards the higher-value item. Alternatively, it might involve continuously directing attention towards the prioritised item and continuously holding it in the FoA, described by Sandry et al. (2020), as an always active or *online* state. Speculatively, paradigms using a highly predictive cue are likely to encourage sole focus on the cued item and possible removal of other items, whereas value studies where reward is not predictive of what will be tested might be more likely to encourage a biased cycling approach, in which lower valuable items do still enter the FoA but less frequently or for shorter amounts of time. Although direct evidence for the effects of attentional refreshing has not always been observed (Bartsch et al., 2018, 2022; Vergauwe et al., 2021; Vergauwe & Langerock, 2023), if viewed as the likelihood of actively retrieving or holding a memory representation in the FoA during maintenance, then it would have some intuitive explanatory value in the context of prioritisation effects.

Attentional refreshing is typically described as a distinct process from verbal rehearsal, that is, overt or covert verbal repetition of some or all the memoranda (Camos et al., 2009). Verbally rehearsing high-priority items represents one modality-specific strategic approach when permitted and relevant to the paradigm. However, most work on value-directed prioritisation implements AS to minimise its contribution (e.g., Allen & Ueno, 2018; Atkinson, Berry, et al., 2018; Hu et al., 2014), and value effects typically survive intact when AS is manipulated (Atkinson, Allen, et al., 2021; Roe et al., 2024). This would demonstrate at least that verbal rehearsal is not the only way of ensuring prioritisation. If a presentation is visuospatial, then spatially oriented rehearsal of occupied locations may also be possible. Spatial processing resources are important in ensuring participants can maintain such information over time (Allen et al., 2023; Hale et al., 1996; Logie & Marchetti, 1991), and participants appear to shift their eye movements towards locations associated with previously presented information during retention (Pearson & Sahraie, 2003; Richardson & Spivey, 2000; Tremblay et al., 2006; though see Loaiza & Souza, 2022). This may help keep representations distinct and effectively bound to a location-based feature map (Kahneman et al., 1992; Treisman & Zhang, 2006), and support attentional refreshing. One possibility is that the locations of high-value objects are fixated for a greater proportion of time during the retention interval.

To summarise, we see value-based prioritisation effects to reflect direct attentional interaction with the environment and mnemonic processes applied during encoding, maintenance, and retrieval. It is likely to reflect a combination of selective attentional priority to stimuli when initially encountered, working memory-based active processing during this attendance, and attentional prioritisation to representations within working memory, post-encoding (though the relative contribution of each of these will vary depending on task context). Central executive control will be required to support prioritisation at each of these points. The result is that high-value information is more likely to be immediately *present* in conscious awareness and available for report, and thus more likely to be successfully retrieved when required (Figure 5). Performance benefits for items of higher value not only on working memory tasks will then emerge, typically on recall accuracy, but also on response time when this is measured (Atkinson et al., 2024; Sandry et al., 2020). This heightened accessibility is further indicated by modelling of performance on a continuous response task, which shows an increased probability of recalling a high-value target (Atkinson et al., 2022). The enhanced response precision observed in this data may reflect selective attention paid during encoding and subsequent maintenance. Finally, the downstream implications of holding information in this state are not limited to recall on an explicit immediate test of performance. Whatever is being held in this state is also more likely to interact with ongoing cognitive processing, influencing where attention is directed, more exposed to influence by subsequent perceptual input and attentional capture, and possibly also more likely to cue retrieval of related information from LTM.

### Different forms of prioritisation

It is useful to contrast research on value-directed prioritisation in working memory with other methods that have been employed when exploring selective attention in this context. One particularly common approach has been to cue an item, usually with a visual prompt (e.g., an arrow or a box highlighting a spatial location) indicating that this item has a high or certain probability of being tested. This has been employed with cues presented before, during, or after target encoding. Research has particularly explored the use of the latter method, applying retro-cues that direct attention towards representations being held in working memory of stimuli that are no longer environmentally present (Souza & Oberauer, 2016). To briefly summarise a sizable literature, items that are indicated as being more likely to be tested are responded to faster and more accurately than uncued items or those that receive a neutral or relatively non-predictive cue. Trade-off patterns like those in value-directed prioritisation have also been observed, with cues shifting around attention but not enhancing overall capacity (Brissenden et al., 2023; Gunseli et al., 2015). We will particularly focus on two key differences between retro-cueing and the focus of the present review, namely, the timing and predictive validity of attentional direction.

The point at which value allocation is provided is likely to be important. Much of the research on value-directed prioritisation has made value information available for encoding (see Figure 1 and Table 1), either at the start of a trial block (e.g., Atkinson, Berry, et al., 2018; Hitch et al., 2018; Hu et al., 2014, 2016, 2023), immediately prior to stimulus presentation (Allen & Ueno, 2018; Atkinson, et al., 2022), or as part of the to-be-encoded display (Sandry & Ricker, 2020; Sandry et al., 2014, 2020). Value-directed prioritisation effects are much smaller when applied retrospectively (Allen & Atkinson, Allen 2021; Brissenden et al., 2023; Jeanneret et al., 2023), in line with Sperling’s (1960) work indicating memory processing constraints following item offset. This also fits with similar findings comparing predictive visual pre-cueing and retro-cueing (Janczyk & Reuss, 2016; Shimi et al., 2014; Souza, 2016), though outcomes are somewhat mixed (Astle et al., 2012; Griffin & Nobre, 2003; Li & Saiki, 2015; Shimi & Scerif, 2017). As encoding is a critical part of any working memory task, examining prioritisation when it is made possible during encoding is important from a theoretical perspective in understanding working memory function, and from an applied perspective as a possible method of supporting efficient, goal-relevant processing. Research using retrospective prioritisation represents undoubtedly important work and continues to generate crucial insights about working memory and attention. However, it omits a major driver of working memory performance, in terms of the mechanisms and strategic approaches that operate during encoding. In addition, retrospective prioritisation is carried out on intrinsically fragile information in working memory, a very different requirement from prioritising externally available information during encoding. Moreover, given that prioritisation is strategic and cognitively demanding (Atkinson, Allen, et al., 2021; Hu et al., 2016), it is possible that asking participants to apply value after encoding and during maintenance represents a demanding secondary task and will interfere with what is being measured. The less reliable benefits of value that are observed may therefore represent both the absence of encoding-based effects and the possible instability and resource cost associated with retrospective application.

A second critical difference is the degree to which attentional direction is predictive of what will be tested. Item reward as allocated in value-directed prioritisation is not predictive of which target will be required at the test phase, and value effects are not dependent on this. Atkinson, Berry, et al. (2018) found that value and test probability both enhanced recall but these factors did not interact, indicating them to be distinct forms of prioritisation. Visual cueing of items, in contrast, has larger and more reliably observed impacts when cues are strongly (e.g., 80% or 100%) predictive of which item will be tested. Predictive cues allow the individual to focus on the cued item while neglecting, inhibiting, or removing others (Liu et al., 2023), whereas allocated reward values that do not predict the test item require the individual to prioritise certain items while still also retaining others from the same encoding event (Jeanneret et al., 2023). Differences in process and representation might then arise between value-based prioritisation and predictive cueing. One impact might be that it shifts the balance between relative protection and vulnerability, as indicated by evidence suggesting that prioritisation induced by predictive retro-cues can protect against perceptual interference (e.g., Makovski & Jiang, 2007; van Moorselaar et al., 2014), whereas high-value items are not protected (and might even be more vulnerable). As noted earlier though, these distinctions are not always clearly observed (e.g., Hu et al., 2023; Vergauwe et al., 2023). For example, Zhang and Lewis-Peacock (2023) compared predictive or reward-based retro-cues and observed similar bias towards perceptual distractors for prioritised and unprioritised information in each case (see also, Rerko et al., 2014).

Prediction and anticipation of the test phase relate to the interesting question of whether there are qualitative or quantitative differences in the underlying representations and processes between prioritised and non-prioritised items, and how effects might specifically impact retrieval. This might in part reflect the mnemonic encoding and maintenance strategies that participants bring to bear on items of differential value (see next section). Impacts emerging during encoding and maintenance ultimately emerge at the test phase of a task when performance is measured. Holding an item in an active state will render it more easily available at test, with impacts on response latency as well as accuracy (e.g., Atkinson et al., 2024; Sandry et al., 2020). One possibility suggested in the context of visual cueing effects is that of a retrieval head-start (Souza & Oberauer, 2016). According to this approach, and as subsequently specified in a diffusion model account (Shepherdson et al., 2018), a retrospective cue that signals which item is likely to be tested ensures a more advanced accumulation of evidence regarding this test-relevant item, thus aiding the decision-making process. Another possibility based on the visual cueing literature is that prioritisation has multiple potential steps, moving from “a task-agnostic mnemonic representation to a task-specific representation that is best suited to guide behavior” (Myers et al., 2017, p. 458). This would imply development of a qualitatively different form of “proceduralised” representation for the prioritised item. These possibilities may be more likely to apply to studies in which visual retro-cues are strongly predictive of what will be tested, thereby allowing sole focus on the cued item, and may prove less directly applicable to the non-predictive value effects that are the primary focus of this review. Nevertheless, the current results may at least partly reflect attention-driven evidence accumulation that supports a more accurate and faster decision at response. It remains to be seen whether predictive and non-predictive forms of prioritisation encourage a quantitative or qualitative shift and how such a shift impacts retrieval.

### Strategy and individual differences

At its core, it is unlikely that there is anything intrinsically special about *value* per se as described here. Instead, value-directed prioritisation represents a tool to encourage the top-down, strategic direction of attention towards certain items in a working memory task. As such, it is ultimately dependent on the individual adhering to the instruction to do so, and we always see variability in the presence and size of benefits between individuals (see Figure 4). It will be valuable to explore what might affect motivation to implement this strategic approach, given that real-world situations will often bring tangible benefits from successfully handling “important” information for subsequent goal-directed action. Relevant dimensions to further explore might include the use of indicative points (as is typically the case in the current work) vs. monetary value (e.g., Zheng et al., 2022) and in-kind rewards, enhanced meaningfulness, and goal relevance of rewards, intrinsic or learned value, and additional motivating factors such as social competition.

Strategically focusing attention on certain items, either based on value or goal relevance, or simply an otherwise arbitrarily selected subset, may be a useful task approach when capacity is stretched or overloaded. Depending on the task, selective encoding of certain stimuli may be a relatively common spontaneously implemented strategy in working memory tasks. For example, Slana Ozimič et al. (2023) identified strategic complexity reduction through the selection of subgroups of stimuli to focus on or ignore as an approach reported by participants in a visual working memory task (see also Atkinson, Baddeley, et al., 2018). Indeed, the starting point for our own research series was the observation by Hu et al. (2014, Experiment 1) that some participants were showing a primacy effect, possibly reflecting a spontaneous strategy of focusing on early sequence items. We therefore initially implemented value manipulations as an experimental method of capturing and controlling this strategic direction of attention. This highlights the importance of considering individual differences and variability in how participants strategically approach a working memory task (e.g., Bartsch et al., 2022; Gonthier, 2021; Logie, 2023; Morrison et al., 2016).

Individual differences in working memory capacity and executive function may be important to consider in this regard. These predict a wide range of broader cognitive abilities and real-world outcomes, with top-down executive-driven attentional control involved in maintaining goal-relevant information and resisting distraction potentially being particularly central to this relationship (Draheim et al., 2022). Relatedly, there is evidence that individuals with low visual working memory capacity (Linke et al., 2011) and low intelligence (Cusack et al., 2009) are more likely to focus on all the items presented during encoding in a working memory task, even beyond capacity limits. In contrast, individuals with higher intelligence and visual working memory capacity are more likely to recognise that this strategy is maladaptive and instead focus on a subset of items when operating beyond capacity limits, which can result in superior performance (Cusack et al., 2009; Linke et al., 2011). Identification and application of appropriate strategy to usefully aid performance in cognitive tasks is also connected to metacognitive awareness (Brown, 1978; Schraw, 2001), which can vary between individuals and develops through childhood (Forsberg et al., 2021). Being able to accurately monitor one’s ongoing task performance and knowing when and how to prioritise important information to avoid cognitive overload, is likely to be a useful metacognitive ability. As a result, there may be reliable variability in the tendency or ability to prioritise with broader predictive value to other related measures.

We have already considered strategically allocated selective attention during encoding and maintenance as a key factor in prioritisation. Depending on task context there might also be scope for active encoding strategies such as elaboration. This approach can enhance subsequent episodic LTM as identified in classic and more recent work using the levels of processing framework (Baddeley & Hitch, 2017; Bartsch et al., 2018; Craik & Tulving, 1975), and elaborative encoding be one method of ensuring high-value items are better encoded into LTM (Cohen et al., 2014). Under the time-, capacity- and resource-limited constraints of a working memory context, deeper elaborative processing might be directed towards more important information, reflecting an active mnemonic component to prioritisation during encoding. The viability and likelihood of such an approach will depend on the individual and the task context; however, elaborative processing is less obviously applicable for simple visual stimuli (e.g., coloured shapes), relative to meaningful verbal memoranda (Siegel & Castel, 2018; Yin et al., 2021). As with verbal rehearsal, elaborative encoding strategies might be a candidate approach in some situations but not the one that is critical to explaining the range of existing findings. It is also worth noting that there is little evidence for the effectiveness of instructed elaboration as an encoding strategy for working memory (Bartsch & Oberauer, 2021; Bartsch et al., 2018), though self-reported elaboration does appear to positively correlate with working memory performance (Bartsch et al., 2022).

### Capacity and order

It is instructive to consider how the paradigms and principles under consideration relate to broader factors of importance in working memory. Two such features heavily in the set of benchmark findings identified by Oberauer et al. (2017) are capacity limits and serial order, and we will briefly discuss each in turn.

As we noted at the start of our review, working memory is defined in part by its limits. What limits might exist in relation to prioritisation? One outstanding issue still to be fully explored concerns the limits on how many items can be prioritised simultaneously. Although studies typically require prioritisation of a single high-value item per trial, a few have examined performance when more than one item in a set is of higher value (Allen & Ueno, 2018; Hitch et al., 2018). In each case, there is evidence for multiple-item prioritisation within a trial, along with performance on low-value items that are above the floor. On a similar note, there is evidence that individuals can prioritise multiple items in response to visual retro-cues (Heuer & Schubö, 2016; Ueno & Allen, under review). Multi-item prioritisation might represent a simultaneous focus on multiple items, or a sequential cycling of these items through selective attention. We assume that resource trade-offs will apply if multiple items are of higher value, depending on the task context. However, aggregation of data across trials in the value-directed studies (Allen & Ueno, 2018; Hitch et al., 2018) somewhat limits the conclusions that can be drawn, and further research is needed.

How might these effects interact with the overall working memory load? Evidence from predictive visual retro-cueing indicates that attentional prioritisation becomes more important when working memory load is high (Shimi & Scerif, 2017). Similarly, both younger and adults showed improved performance overall when encouraged to focus on a (self-selected) subset of items rather than the whole array, particularly when the set size was higher (Atkinson, Baddeley, et al., 2018). Our assumption is that strategic prioritisation based on item importance/value would likewise become a more useful approach when working memory is otherwise stretched to or beyond capacity. We have some evidence for this in a developmental context, in that children only showed a value effect when remembering simultaneous arrays of four and not three items (Atkinson et al., 2019). This remains to be systematically explored, though observed value effects tend to be larger and more reliable when using four-item sequences (e.g., Hitch et al., 2018) rather than three items (Allen et al., 2021), at least for young adults.

A second common theme in short-term and working memory research is that of temporal and ordinal coding. Information can be retained in serial order in working memory, and accuracy varies with position in a sequence, with improved recall for early (primacy) and late (recency) sequence items (Oberauer et al., 2017). As already discussed, the recency boost has been a strong focus in our work, reflecting an automatic component that is distinct from strategic top-down control. Regarding prioritisation, value has often been allocated based on ordinal position in a sequence, alongside other methods (see Table 1 and Figure 1). However, the response measure is typically item-focused, using tasks such as single-item cued recall (Allen & Ueno, 2018; Hu et al., 2014), recognition (Atkinson et al., 2024), or precision-based continuous response tasks (Atkinson et al., 2022; Hu et al., 2023) that do not explicitly require order memory. The exceptions to this are two studies using serial recall where both item and order are emphasised (Atkinson, Allen, et al., 2021; Roe et al., 2024). In discussing prioritisation effects, we would note that we use “items” as shorthand to refer to information in more general terms, and it is not intended as a contrast with order. We see no reason why strategic priority could not be equally applied to item or order information, and it would be worthwhile to examine the impacts of value on order memory.

### Extending to LTM

We know that operations applied during working memory can influence subsequent retention and retrieval in LTM tasks (Camos & Portrat, 2015; Cotton & Ricker, 2021). To what extent do prioritisation effects extend beyond working memory into LTM? There is a sizable literature on value-directed remembering in episodic LTM (Knowlton & Castel, 2022), and some evidence that items cued in working memory tasks are better remembered later (Jeanneret et al., 2023; Reaves et al., 2016; Strunk et al., 2019). However, value in working memory does not appear to consistently translate into lasting benefits on later unexpected tests. On one hand, Sandry et al. (2020) observed a small effect of working memory value on a delayed free recall task under certain conditions. In contrast, Jeanneret et al. (2023) saw little advantage for retro-cued or retro-valued items on a delayed recognition test, and Atkinson et al. (2024) found no consistent evidence that working memory value influenced performance on a delayed recognition task. It will be theoretically and practically useful to establish to what extent working memory value effects are ephemeral or more long-lasting, and what might underlie the apparent differences with the reliable effects seen in episodic LTM.

### Practical applications

A major part of the multicomponent approach to working memory as introduced by Baddeley and Hitch (1974) and subsequently continued in myriad ways has been its impact in applied contexts, as covered in part by the recent collection of chapters edited by Logie et al. (2023). Given the practical importance of working memory and attentional control (Draheim et al., 2022), it should prove worthwhile to explore how we might productively extend the principles of prioritisation to applied contexts. We have set out evidence that strategic prioritisation based on task “value” is possible across a range of tasks and domains, not only during young adulthood but also at points in the lifespan when working memory and cognitive control are otherwise relatively limited. Given the apparent simplicity and generalisability of the core phenomenon, strategic prioritisation may therefore offer useful ways forward in supporting the efficiency of these key components of cognition in the service of task goals when they are required in real-world contexts beyond the lab.

We see top-down strategic prioritisation as being important in any situation that requires keeping track of, thinking about, and reacting to information in complex scenarios where working memory capacity is otherwise stretched or overloaded. For example, an air traffic controller (using a classic example from applied cognition) must monitor, manage, and respond to flight details, and a hospital nurse often needs to listen to a patient’s list of symptoms and prioritise important details. Within an educational context, a child in a classroom is often required to try and follow lengthy and detailed instructions from a teacher (Allen et al., 2023; Atkinson, Allen, & Waterman, 2021; Gathercole et al., 2006), and in turn, the teacher needs to monitor and manage the behaviours and demands of multiple children while delivering a planned learning activity. In each case, task performance within demanding and dynamically changing contexts can be improved by selectively applying attentional focus to prioritise holding important details in working memory. Developing guidance and training in metacognitive awareness and cognitive control might offer promising ways forward in helping individuals make the most of their otherwise highly constrained working memory capacity.

## Conclusion

The apparent close symbiotic relationship between working memory and attentional control is an important and growing focus of the field. In exploring how extrinsic and intrinsically controlled attention influence working memory, we identified a possible spontaneously applied strategy for prioritisation that some participants employ (Hu et al., 2014), which might serve to support task performance within a limited-capacity system. Subsequent work has harnessed this through value-directed prioritisation, an effective tool for understanding the nexus between working memory, attentional control, and strategy use across a broad range of different task contexts and populations. It has helped bridge the gap between our broad multi-component framework and the literature on attentional control and working memory that has emerged over the last 20 years, as well as highlighting ways in which different theoretical perspectives on working memory might share some common ground. There is still much to establish regarding how prioritisation might vary with changes in task demands, and what this might tell us about working memory and selective attention. More work is required to establish the boundary conditions on the core phenomena (i.e., trade-offs between a high-value benefit and a low-value cost, with no overall change in performance), the constraints on prioritisation, and how these might be determined by strategic control, availability of executive control resources, and perceptual interference.
